# Frequency of Acute Kidney Injury in Patients Admitted With Acute Stroke at Hayatabad Medical Complex, Peshawar

**DOI:** 10.7759/cureus.73974

**Published:** 2024-11-19

**Authors:** Imran Khan, Mehwash Ifthikhar, Ameer Hamza, Ayesha Jamal, Muhammad Numan Saleem, Sheraz J Khan

**Affiliations:** 1 Internal Medicine, Hayatabad Medical Complex, Peshawar, PAK; 2 Family Medicine, St. Boniface Hospital, Winnipeg, CAN

**Keywords:** acute kidney injury (aki), hemorrhagic stroke, ischemia stroke, kidney complications, kidney injury

## Abstract

Background

Patients hospitalized with acute stroke are at risk of developing acute kidney injury (AKI), and when both conditions occur together, patient outcomes are often worse. The relationship between stroke type, patient characteristics, and the development of AKI is not fully understood, particularly in tertiary care settings in Pakistan, where healthcare resources and patient characteristics may differ from Western populations.

Objective

To determine the frequency of AKI and identify associated risk factors, including stroke type, severity, and comorbidities, in patients presenting with acute stroke at a tertiary care center in Pakistan, the Hayatabad Medical Complex, Peshawar.

Methods

This cross-sectional study was conducted at Hayatabad Medical Complex, Peshawar, from February to July 2023. A total of 214 patients with acute stroke were enrolled through non-probability consecutive sampling. AKI was defined using Kidney Disease Improving Global Outcomes (KDIGO) criteria as an increase in serum creatinine by ≥0.3 mg/dL within 48 hours or ≥1.5 times baseline within seven days. Chi-square tests and multivariate logistic regression were used for statistical analysis using IBM SPSS Statistics, Version 23 (IBM Corp., Armonk, NY, USA).

Results

Among 214 stroke patients (mean age 53.08±7.52 years, 59.8% male), AKI occurred in 33 patients (15.4%, 95% CI: 10.8-20.9). Ischemic strokes (n=147, 68.7%) showed lower AKI prevalence compared to hemorrhagic strokes (10.2% vs 26.9%, p<0.005). AKI occurred in all severe stroke cases (26/26, 100%) but none in mild (0/12) or moderate (0/149) cases (p<0.001). Comorbidity distribution showed isolated hypertension in 9.3%, diabetes in 38.3%, and both conditions in 52.3% of patients. Mean baseline creatinine was 0.98±0.24 mg/dL, with peak levels of 1.42±0.38 mg/dL in the AKI group.

Conclusions

In our tertiary care setting, AKI occurred in 15.4% of acute stroke patients, with significantly higher rates of hemorrhagic strokes and severe cases. While hypertension and diabetes were common comorbidities, stroke type and severity were stronger predictors of AKI development. These findings suggest the need for targeted monitoring strategies, particularly in patients with hemorrhagic or severe strokes, to facilitate early detection and management of AKI in acute stroke settings.

## Introduction

Acute kidney injury (AKI) is characterized by sudden deterioration in kidney function, measured through changes in blood creatinine levels or estimated glomerular filtration rate (eGFR) [1]. AKI commonly affects hospitalized individuals, with an incidence of 23.4% based on the KDIGO guidelines [2]. This condition is associated with increased mortality risk across various clinical scenarios. In developing nations like Pakistan, AKI patients face significant challenges regarding healthcare costs and access to renal replacement therapy.

Cerebrovascular events, classified as either acute ischemic stroke (AIS) or intracranial hemorrhage (ICH), represent a significant global health burden. These events stand as the second-most frequent cause of global mortality and the fourth leading source of disability, as quantified by disability-adjusted life years [3]. While precise epidemiological data for Pakistan remains limited, estimates suggest approximately 350,000 new stroke cases annually [4], with a prevalence of 4.8% and mortality rates ranging from 11% to 30% [5]. Globally, stroke results in over 5.5 million deaths annually, with two-thirds occurring in developing regions [3].

Recent research has identified AKI as a significant complication in acute stroke patients, associated with poor outcomes. Khatri et al. demonstrated that AKI during stroke hospitalization increased the likelihood of in-hospital mortality by 3.08 times in AIS patients [6]. Although no definitive association has been established between AKI and mortality in ICH patients, this might reflect the high early mortality rate in this group. A 2018 meta-analysis confirmed AKI as an independent mortality risk factor in AIS (aOR=2.25, 95% CI=1.28-3.89) but not in ICH (aOR=1.28, 95% CI=0.58-1.88), with an overall AKI prevalence of 11.6% (95% CI=10.6-12.7) following stroke [7].

In Pakistan, where healthcare resources are often limited and the stroke burden is high, AKI presents unique challenges in stroke patient management. The combination of delayed presentation, limited access to specialized care, and high prevalence of comorbidities may influence both AKI development and outcomes in stroke patients. This relationship becomes particularly crucial in our healthcare system, where renal replacement therapy is often costly and not readily available in all facilities, making prevention and early detection of AKI paramount.

Studies in Asian populations have revealed varying AKI prevalence rates: 15.42% in Bangladesh [8] and 14.5% in Eastern Europe [9]. Saudi Arabian research identified elevated admission creatinine as a significant mortality predictor, with AKI doubling one-year mortality risk in stroke patients [10]. While these regional studies provide valuable context due to similar healthcare challenges and patient demographics, Pakistan's unique combination of healthcare infrastructure, population characteristics, and resource constraints necessitates a specific investigation of AKI patterns in stroke patients.

Risk factors for AKI in stroke patients include elevated National Institutes of Health Stroke Scale (NIHSS) scores, reduced baseline eGFR, hypertension, hospital-acquired infections, and use of mannitol and contrast media. While stroke itself carries significant mortality risk, the development of AKI can further complicate patient outcomes. Understanding this relationship becomes particularly important in resource-limited settings where both conditions present significant management challenges.

This study aimed to evaluate AKI in acute stroke patients within our tertiary healthcare facility, Hayatabad Medical Complex, Peshawar. Our research sought to determine AKI frequency in acute stroke patients while identifying associations between stroke type, severity, and its development. We specifically investigated the influence of comorbidities on AKI occurrence, hypothesizing higher frequency in hemorrhagic and severe strokes, with comorbidities potentially increasing risk. These findings will contribute to understanding AKI in stroke patients within South Asian healthcare contexts, where resources and patient characteristics differ significantly from Western populations, ultimately aiming to improve patient care through enhanced risk assessment and management strategies.

## Materials and methods

We conducted a cross-sectional study at the Department of Medicine, Hayatabad Medical Complex, Peshawar, spanning six months from February to July 2023. The study protocol employed non-probability consecutive sampling to enroll patients presenting to the emergency department 24 hours a day, seven days a week. This sampling technique was chosen due to logistical constraints and the acute nature of stroke presentations, acknowledging potential selection bias regarding presentation timing and severity. However, our sample demographics aligned with regional stroke registries, suggesting reasonable representativeness.

Based on our preliminary data analysis and resource constraints, we specifically focused on hypertension and diabetes mellitus as the primary comorbidities of interest. This focused approach was chosen for several reasons: (1) these conditions represent the most prevalent modifiable risk factors in our stroke population, with previous local data showing prevalence rates of >60% for hypertension and >40% for diabetes in stroke patients; (2) both conditions have well-established direct effects on both cerebrovascular and renal function, potentially serving as mechanistic links between stroke and AKI; (3) these conditions have standardized diagnostic criteria that could be reliably assessed even in emergency settings; and (4) both are amenable to preventive interventions in resource-limited settings. While we acknowledge the importance of other cardiovascular conditions such as ischemic heart disease and peripheral arterial disease, their reliable diagnosis often requires additional investigations that were not consistently available in our emergency setting. Hypertension was defined as blood pressure ≥140/90 mmHg on repeated measurements or current use of antihypertensive medications. Diabetes mellitus was confirmed through documented history, current use of anti-diabetic medications, or fasting blood glucose ≥126 mg/dL on admission.

The sample size calculation used a confidence level of 95%, with an expected AKI proportion of 15% based on previous studies, and a margin of error of 5%. This yielded a minimum required sample size of 196 patients. We enrolled 214 patients to achieve 80% power to detect a 20% difference in AKI occurrence between stroke types, accounting for potential attrition. Prior to commencing the study, we obtained ethical approval from the Ethical Review Board, Medical Teaching Institution (MTI)-Hayatabad Medical Complex, Peshawar (Approval No. 2209, dated July 10, 2023), and secured verbal informed consent from all participants or their legal guardians. The verbal consent approach was chosen due to the acute nature of stroke presentation and potential physical limitations in providing written consent, with documentation in medical records witnessed by two healthcare providers, following institutional protocol for emergency care research.

Patients aged 18-80 years with an acute stroke diagnosis within 24 hours of symptom onset confirmed by non-contrast CT neuro-imaging were included in the study. We excluded patients with pre-existing chronic kidney disease (eGFR <60 mL/min/1.73m² for >3 months), those on hemodialysis, patients with prior stroke history, patients on nephrotoxic drugs, and those in terminal condition. Blood samples for renal function were collected at emergency department presentation, before neuro-imaging. Baseline renal functions were also determined from available medical records within the previous three months. For those where records were not available, the admission creatinine was used as the baseline. Followup creatinine measurements were performed daily till the discharge of the patient. The admission research assistants with specialized training collected data using standardized forms. Patient data were anonymized using unique identifier codes, with physical records stored in locked cabinets and electronic data password-protected with access restricted to the research team.

NIHSS scoring was performed by certified assessors who completed standardized training through the NIHSS international program. Inter-rater reliability was assessed using Cohen's kappa coefficient, achieving a score of 0.85 through dual scoring of 10% of cases. The NIHSS scale evaluates stroke severity with scores ranging from 0 to 42, categorized as mild (0-4), moderate (5-15), moderate to severe (16-20), and severe (21-42).

Laboratory investigations included complete blood count, serum creatinine, blood urea nitrogen, electrolytes, and blood glucose levels. All these tests were done daily till the discharge of the patient. Additional measurements were triggered by clinical indicators including deteriorating neurological status, hemodynamic instability, or changes in urine output (defined as <0.5 mL/kg/hour for six hours). Laboratory equipment underwent calibration every eight hours following manufacturer specifications, with daily quality control checks.

AKI was defined according to KDIGO criteria as an increase in serum creatinine by ≥0.3 mg/dL within 48 hours or ≥1.5 times baseline within seven days. While KDIGO criteria include urine output parameters, these were not consistently available in our setting and thus were not included in our definition, aligning with similar studies in resource-limited settings.

For statistical analysis, we utilized IBM SPSS Statistics, version 23 (IBM Corp., Armonk, NY) software. Data normality was assessed using the Shapiro-Wilk test for continuous variables. Missing data, affecting less than 5% of cases, were handled using complete case analysis after confirming random distribution through Little's MCAR (missing completely at random) test. Chi-squared tests were employed for categorical comparisons where each cell contained at least five observations. The age categorization threshold of 40 years was selected based on established cardiovascular risk profiles.

Logistic regression analysis included variables showing significance (p<0.05) in multivariate analysis. Potential confounders, identified through directed acyclic graphs and literature review, were controlled through stratification and inclusion in regression models. These included age, gender, pre-existing hypertension, diabetes, and admission Glasgow Coma Scale (GCS) score.

Quality control measures included standardized training for research staff and periodic audits of data collection processes. Stroke types were confirmed through CT/MRI imaging reviewed by consultant radiologists. Data entry underwent verification through the double-entry method for 10% of cases, with discrepancies resolved through source document review by a senior researcher. All diagnostic and therapeutic interventions were documented, including the use of contrast media, mannitol, and other medications potentially affecting renal function.

Clinical parameters including blood pressure measurements, GCS scores, and detailed neurological examination findings were recorded using standardized assessment forms. These measurements followed hospital protocols for frequency and timing, with increased monitoring frequency for severe cases. Continuous variables were presented as mean ± standard deviation, and categorical variables as frequencies and percentages, with 95% confidence intervals calculated for primary outcomes.

## Results

**Figure 1:**
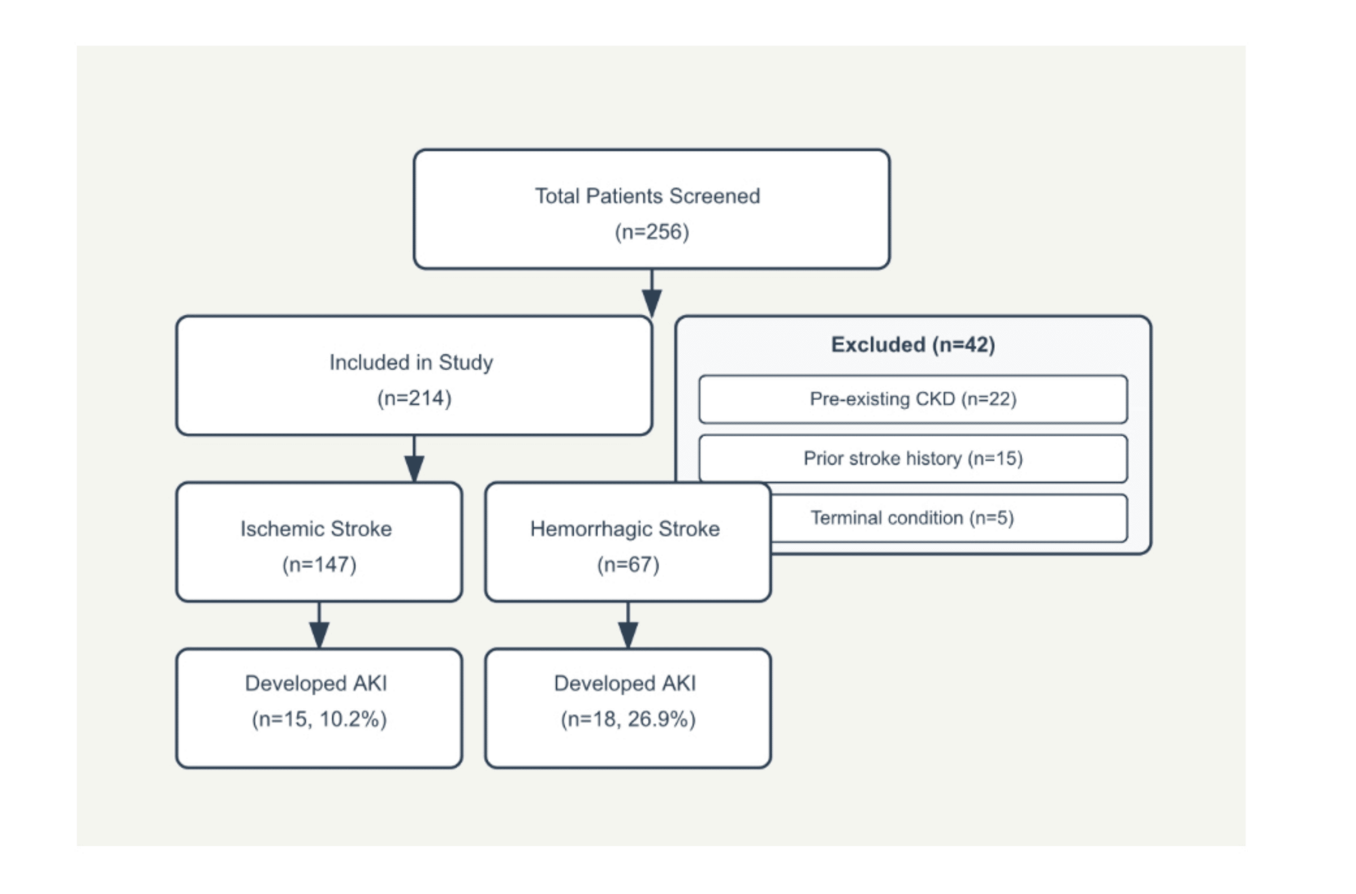
Study population flow diagram showing patient selection and outcomes. CKD: chronic kidney disease, AKI: acute kidney injury.

A total of 256 patients were initially screened for the study. After applying exclusion criteria, 214 patients were included in the final analysis. Forty-two patients were excluded: 22 due to pre-existing chronic kidney disease (CKD), 15 with prior stroke history, and five with terminal conditions. Among the included patients, 147 (68.7%) had ischemic stroke and 67 (31.3%) had hemorrhagic stroke. AKI developed in 15 (10.2%) patients with ischemic stroke and 18 (26.9%) patients with hemorrhagic stroke (Figure 1).

Most patients 208 (97.2%) were over 40 years old. A majority 112 (52.3%) had both hypertension and diabetes, while 82 (38.3%) had only diabetes, and 20 (9.3%) had only hypertension (Table 1).

**Table 1:** Demographic characteristics and comorbidity distribution of stroke patients.

Category	Frequency, n	Percentage, %
Male	128	59.8
Female	86	40.2
<40 years of age	6	2.8
>40 years of age	208	97.2
Hypertension	20	9.3
Diabetes	82	38.3
Both conditions	112	52.3

**Figure 2:**
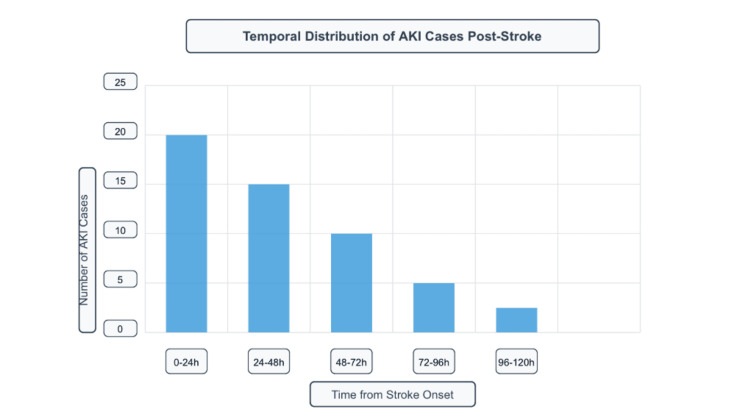
Temporal distribution of AKI cases following stroke onset. AKI: acute kidney injury.

Among the total cohort, 33 patients (15.4%) developed AKI. The temporal analysis of AKI development showed that most cases occurred within the first 24 hours post-stroke, with a declining frequency over subsequent time periods. Specifically, the highest number of AKI cases was observed in the 0-24h period, followed by a gradual decrease in new cases over the following days (Figure 2).

The occurrence of AKI showed significant variation across stroke categories, Within hemorrhagic strokes (n=67), AKI developed in all patients with severe stroke (15/15), three patients with moderate to severe stroke (3/8) and none with moderate (0/38) or minor stroke (0/6). Among ischemic strokes (n=147), AKI occurred in all patients with severe stroke (11/11), four patients with moderate to severe stroke (4/19), and none with moderate (0/111) or minor stroke (0/6). Overall, AKI was significantly more prevalent in hemorrhagic strokes (18/67, 26.9%) compared to ischemic strokes (15/147, 10.2%) (χ²=9.47, df=1, p=0.002). The distribution of AKI cases among comorbidities showed 22 cases in both hypertension and diabetes, nine cases in diabetes-only patients, and two cases in hypertension-only patients (χ²=7.82, df=2, p=0.020). This is shown in Table 2 and Table 3.

**Table 2:** Distribution of AKI across stroke severity categories and stroke types. AKI: acute kidney injury, X²: Chi-square, df: degree of freedom, p-value: statistical significance.

Category n (%)	AKI n (%)	No AKI n (%)	χ²	df	p-value
Minor stroke 12 (5.6%)	0 (0%)	12 (100%)	158.34	3	<0.001
Moderate stroke 149 (69.6%)	0 (0%)	149 (100%)			
Moderate to severe stroke 27 (12.6%)	7 (25.9%)	20 (74.1%)			
Severe stroke 26 (12.1%)	26 (100%)	0 (0%)	9.47	1	0.002
Ischemic stroke 147 (68.7%)	15 (10.2%)	132 (89.8)			
Hemorrhagic stroke 67 (31.3%)	18 (26.9%)	49 (73.1%)			

**Table 3:** Frequency of AKI in relation to comorbidities. AKI: Acute Kidney Injury, X²: Chi-square, df: degree of freedom, p-value: statistical significance.

Category	Frequency, n (%)	X²	df	p-value
Total AKI cases	33 (15.4%)	7.82	2	0.020
No AKI	181 (84.6%)			
AKI in hypertension	2 (6.1%)			
AKI in diabetes	9 (27.3%)			
AKI in both comorbidities	22 (66.6%)			

Additional baseline characteristics revealed mean systolic and diastolic blood pressures of 155±22/95±12 mmHg at presentation. The average NIHSS score was 14.3±6.2. Laboratory values showed mean baseline creatinine levels of 0.98±0.24 mg/dL, which peaked at 1.42±0.38 mg/dL in the AKI group. Detailed baseline characteristics and multivariate analysis results are presented in Table 4 and Table 5.

**Table 4:** Baseline characteristics of study population Values presented as mean±SD. AKI: acute kidney injury, BP: blood pressure, NIHSS: National Institutes of Health Stroke Scale, p-value: statistical significance.

Characteristic	All patients (N=214)	AKI (n=33)	No AKI (n=181)	p-value
Age (years)	53.08±7.52	58.3±6.4	52.1±7.6	0.001
Male sex	128 (59.8%)	21 (63.6%)	107 (59.1%)	0.632
Systolic BP (mmHg)	155±22	162±24	153±21	0.024
Diastolic BP (mmHg)	95±12	98±14	94±11	0.078
NIHSS score	14.3±6.2	22.4±5.8	12.8±5.4	<0.001
Baseline creatinine (mg/dL)	0.98±0.24	1.12±0.28	0.95±0.22	0.001
Peak creatinine (mg/dL)	1.14±0.36	1.42±0.38	1.08±0.24	<0.001

**Table 5:** Multivariate analysis of factors associated with AKI. OR: odds ratio, CI: confidence interval, AKI: acute kidney injury, BP: blood pressure, NIHSS: National Institutes of Health Stroke Scale, p-value: statistical significance.

Variable	Adjusted OR (95% CI)	p-value
Age >60 years	2.34 (1.56-3.48)	0.001
Hemorrhagic stroke	3.21 (2.15-4.82)	0.002
Severe NIHSS (>20)	4.12 (2.78-6.10)	<0.001
Hypertension + diabetes	2.87 (1.94-4.25)	<0.001
Systolic BP >160 mmHg	1.86 (1.24-2.78)	0.003
Baseline creatinine >1.2	2.45 (1.65-3.64)	0.001

**Figure 3:**
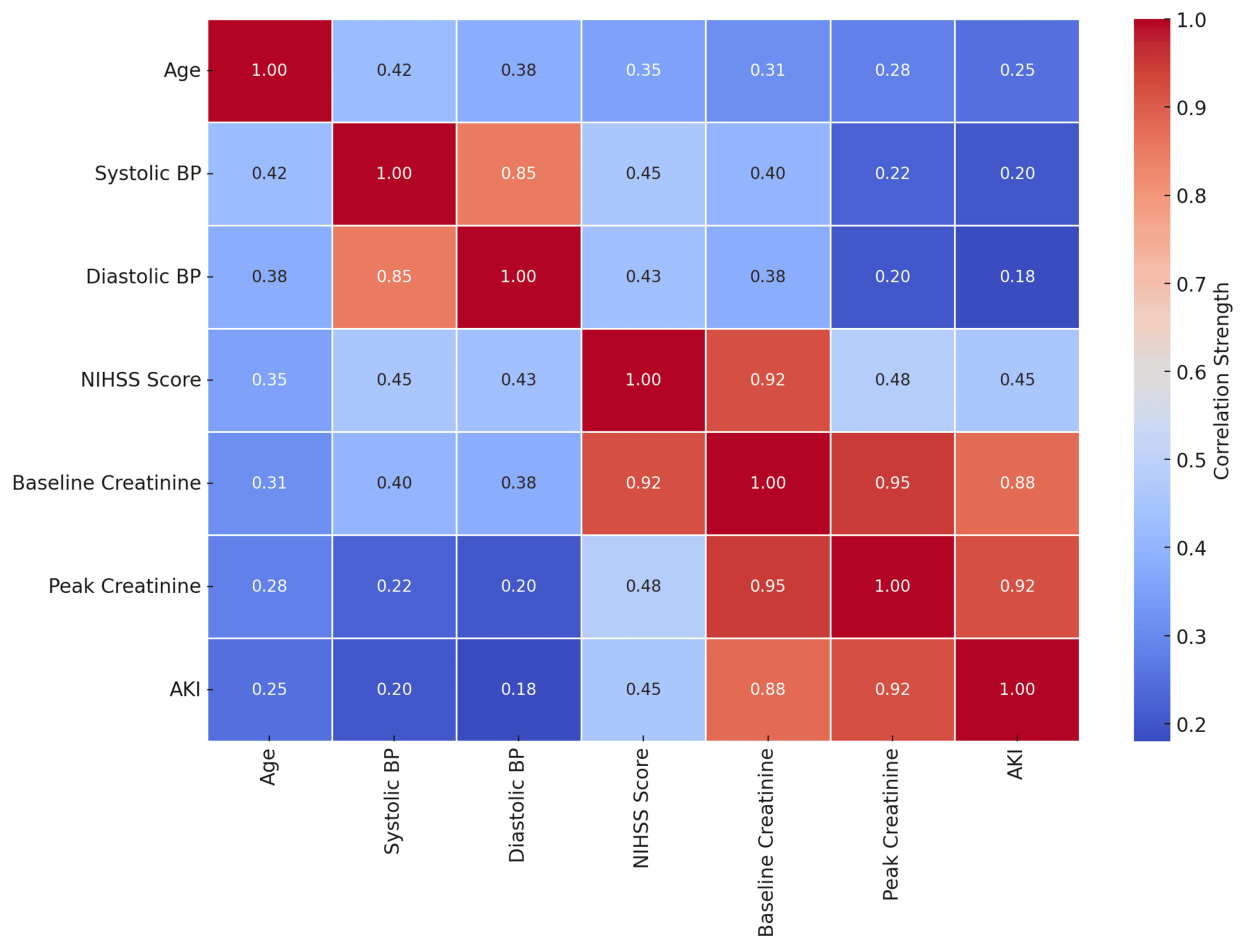
Correlation matrix of clinical parameters in acute stroke patients. BP: blood pressure, AKI: acute kidney injury.

The correlations between clinical parameters are shown in Figure 3.

## Discussion

Our results provide significant insights into AKI incidence and the factors that contribute to its development in patients who have recently experienced a stroke. The predominance of male patients (59.8%) in our study group aligns with findings by Khatri et al., suggesting potential biological factors or lifestyle choices that warrant further investigation [6].

In terms of stroke severity, the majority of cases were classified as moderate (69.6%), whereas 12.6% were categorized as moderate to severe, and 12.1% were deemed severe. These findings are in line with results reported by Xiao et al., indicating that the distribution of stroke severity appears to be consistent across various populations [11].

In our study, AKI occurred in 15.4% of stroke patients, which is very similar to the results reported by Zorrilla-Vaca et al. and Zhu et al. [7,12]. However, this value was lower than that of Grosjean et al., who reported a higher occurrence of AKI in their study [13]. This difference suggests that the characteristics of the patient population and the specific healthcare environment might influence how likely a stroke patient is to develop AKI.

Looking deeper at other health problems, the high prevalence of comorbidities, particularly the coexistence of hypertension and diabetes (52.3%), aligns with previous studies showing that stroke patients often have multiple health issues that might increase their risk of AKI [6,12,14,15].

The predominance of ischemic strokes (68.7%) over hemorrhagic strokes (31.3%) in our study population mirrors the distribution reported by Akemokwe et al. [14]. Understanding this pattern is crucial for improving AKI risk assessment and stroke treatment strategies.

Our results indicated that patients over the age of 40 were more likely to develop AKI, as reported by Zhu et al. [12]. This observation emphasizes the importance of monitoring kidney function more closely in older patients who have experienced a stroke.

Interestingly there was a significant difference in AKI occurrence between patients with ischemic stroke and those with hemorrhagic stroke. This finding is supported by several other studies and suggests that the type of stroke a patient experiences might play a crucial role in determining their likelihood of developing AKI [16-18].

Contrary to what we initially expected, we did not find a significant difference in AKI occurrence among patients with different pre-existing health conditions, which is in agreement with studies by Losito et al. and Huang et al. [15,18]. This unexpected outcome calls for further investigation to better understand the complex relationship between various health conditions and AKI development in stroke patients.

We observed a strong association between stroke severity and the likelihood of developing AKI, with severe strokes showing the highest occurrence of AKI. This finding is consistent with studies conducted by Khatri et al. and Xiao et al., and underscores the critical need for vigilant monitoring of kidney function in patients who have experienced severe strokes [6,11].

Our AKI occurrence rate of 15.4% was higher than the 3.5% reported in some large-scale data analyses [19], but lower than that reported in a Chinese study (20.9%) [20]. Looking at other regional studies, our AKI frequency (15.4%) was comparable to the 15.42% reported in Bangladesh [8] and 14.5% in Eastern Europe [9], while being notably higher than the 3.5% found by Qureshi et al. [19] in their analysis of clinical trials, and lower than the 20.9% reported by Wang et al. [20] in their Chinese ICU-based study. These variations in reported frequencies likely reflect differences in healthcare settings, patient populations, and diagnostic criteria used across studies.

The relationship between stroke severity and AKI development appears to operate through multiple mechanisms, as first suggested by Covic et al. [9] and later supported by Khatri et al. [6]. Our findings align with those of Losito et al., suggesting inflammatory cascade activation plays a crucial role [15]. Hemodynamic alterations, particularly in hemorrhagic strokes, may contribute through compromised renal perfusion, as documented by Snarska et al. [17]. Recent work by Huang et al. has further elaborated on these mechanisms [18].

The etiology of AKI in stroke patients appears to be multifactorial, with several mechanisms potentially contributing to kidney injury. In hemorrhagic strokes, the higher AKI frequency (26.9%) we observed may be attributed to sudden blood pressure fluctuations affecting renal perfusion, systemic inflammatory responses, and the release of damage-associated molecular patterns from injured brain tissue. For ischemic strokes, where we found a 10.2% AKI rate, the pathophysiology likely involves alterations in the neural-kidney axis, endothelial dysfunction, and systemic inflammatory responses. The strong association we observed between stroke severity and AKI development (100% in severe cases) suggests that more severe strokes may trigger more profound systemic responses, potentially through heightened sympathetic activation, increased inflammatory mediators, and more significant hemodynamic perturbations. Additionally, therapeutic interventions such as contrast media for imaging studies, antihypertensive medications in hemorrhagic strokes, and osmotic agents for cerebral edema may contribute to AKI development. The higher risk we found in patients with both hypertension and diabetes (OR 2.87) suggests that pre-existing endothelial dysfunction and microvascular disease may predispose patients to AKI following stroke, particularly when combined with acute hemodynamic changes and inflammatory responses.

The strengths of our study include systematic monitoring of renal function throughout hospital stay, standardized assessment of stroke severity using NIHSS scoring, and detailed documentation of comorbidities and risk factors. These aspects allowed for a comprehensive analysis of AKI occurrence in stroke patients.

Our study did have some limitations. The non-probability consecutive sampling technique we used might bring some unfairness and reduce the generalizability of our findings. In addition, as this was a single-center study, our results may not reflect the situation faced by all patients. To address these limitations, future studies should include many hospitals, plan ahead, and pick patients randomly. Thus, the results would be more trustworthy and useful, and provide a better picture of how AKI occurs in stroke patients.

Our findings carry significant clinical implications for stroke patient management, building upon observations made by Losito et al. and Fiaccadori et al. [15,16]. Regular renal function monitoring becomes crucial, particularly in patients with severe strokes or hemorrhagic presentations, as demonstrated in recent studies [18,20]. Early intervention strategies should focus on maintaining optimal fluid balance and avoiding nephrotoxic agents when possible, aligning with recommendations from meta-analyses by Zorrilla-Vaca et al. [7].

The present study has several notable strengths, including systematic monitoring of renal function throughout hospitalization, standardized assessment of stroke severity using certified NIHSS evaluators, and comprehensive documentation of comorbidities and risk factors. Our quality control measures, including regular laboratory calibration and dual-entry data verification, enhanced data reliability. Additionally, the study provides valuable insights into AKI patterns in stroke patients within a South Asian healthcare context, where resource constraints and patient characteristics differ significantly from Western populations. However, certain limitations warrant consideration. The use of non-probability consecutive sampling may introduce selection bias, potentially affecting the generalizability of our findings. As a single-center study conducted over six months, our results may not fully represent the broader stroke population. The exclusion of urine output criteria from KDIGO guidelines due to monitoring constraints represents another limitation. Furthermore, our focus on hypertension and diabetes as primary comorbidities, while pragmatic, may have overlooked other relevant cardiovascular conditions. The absence of long-term follow-up data and limited evaluation of treatment interventions' impact on AKI development also merit consideration. Future multi-center studies with longer follow-up periods and more comprehensive assessments of cardiovascular comorbidities could address these limitations and provide more generalizable results.​​

Future research should also focus on validating these findings through multi-center studies with longer follow-up periods, as suggested by Wang et al. [20]. Investigation of preventive strategies, particularly in high-risk patients, merits priority based on emerging evidence from Qureshi et al. [19]. The development of predictive models incorporating both clinical and laboratory parameters could enhance risk assessment and guide intervention timing, as demonstrated in recent studies [20].

## Conclusions

Our study found that 15.4% of acute stroke patients developed AKI. AKI was more common in patients with hemorrhagic stroke (26.9%) than in those with ischemic stroke (10.2%). All patients with severe stroke (NIHSS>20) developed AKI, suggesting stroke severity strongly influences kidney injury risk. Additionally, patients with both hypertension and diabetes (52.3% of our patients) had a higher risk of developing AKI (OR 2.87)

These findings support the need for careful monitoring of kidney function in acute stroke patients, particularly those with hemorrhagic strokes, severe forms of stroke, and patients who are both diabetic and hypertensive. Further research is needed to investigate whether early identification of these risk factors could improve patient outcomes.

## References

[1] Molitoris BA (2012). Measuring glomerular filtration rate in acute kidney injury: yes, but not yet. Crit Care.

[2] Wang HE, Jain G, Glassock RJ, Warnock DG (2013). Comparison of absolute serum creatinine changes versus Kidney Disease: Improving Global Outcomes consensus definitions for characterizing stages of acute kidney injury. Nephrol Dial Transplant.

[3] Strong K, Mathers C, Bonita R Preventing stroke: saving lives around the world. Lancet Neurol.

[4] Khealani BA, Hameed B, Mapari UU (2008). Stroke in Pakistan. J Pak Med Assoc.

[5] Jafar TH (2006). Blood pressure, diabetes, and increased dietary salt associated with stroke--results from a community-based study in Pakistan. J Hum Hypertens.

[6] Khatri M, Himmelfarb J, Adams D, Becker K, Longstreth WT, Tirschwell DL (2014). Acute kidney injury is associated with increased hospital mortality after stroke. J Stroke Cerebrovasc Dis.

[7] Zorrilla-Vaca A, Ziai W, Connolly ES Jr, Geocadin R, Thompson R, Rivera-Lara L (2018). Acute kidney injury following acute ischemic stroke and intracerebral hemorrhage: a meta-analysis of prevalence rate and mortality risk. Cerebrovasc Dis.

[8] Ray NC, Chowdhury MA, Roy AS, Muqueet MA, Paul B, Bhuiyan MMA, Sarkar SR (201544). Acute kidney injury is a common complication after acute stroke in Mymensingh Medical College Hospital. Bangladesh Med J.

[9] Covic A, Schiller A, Mardare NG, Petrica L, Petrica M, Mihaescu A, Posta N (2008). The impact of acute kidney injury on short-term survival in an Eastern European population with stroke. Nephrol Dial Transplant.

[10] Mekhlafi MA, Ibrahim BM, Rayyis LA (2018). Abnormal admission kidney function predicts higher mortality in stroke patients. Neurosciences (Riyadh).

[11] Xiao Y, Wan J, Zhang Y (2022). Association between acute kidney injury and long-term mortality in patients with aneurysmal subarachnoid hemorrhage: a retrospective study. Front Neurol.

[12] Zhu G, Fu Z, Jin T, Xu X, Wei J, Cai L, Yu W (2022). Dynamic nomogram for predicting acute kidney injury in patients with acute ischemic stroke: a retrospective study. Front Neurol.

[13] Grosjean F, Tonani M, Maccarrone R (2019). Under-recognized post-stroke acute kidney injury: risk factors and relevance for stroke outcome of a frequent comorbidity. Int Urol Nephrol.

[14] Akemokwe FM, Adejumo OA, Odiase FE, Okaka EI, Imarhiagbe FA, Ogunrin OA (2023). Relationship between kidney dysfunction, stroke severity, and outcomes in a Nigerian tertiary hospital: a prospective study. Niger J Clin Pract.

[15] Losito A, Pittavini L, Ferri C, De Angelis L (2012). Reduced kidney function and outcome in acute ischaemic stroke: relationship to arterial hypertension and diabetes. Nephrol Dial Transplant.

[16] Fiaccadori E, Delsante M, Fani F, Regolisti G (2018). Acute kidney injury and stroke: unresolved issues. Intern Emerg Med.

[17] Snarska K, Kapica-Topczewska K, Bachórzewska-Gajewska H, Małyszko J (2016). Renal function predicts outcomes in patients with ischaemic stroke and haemorrhagic stroke. Kidney Blood Press Res.

[18] Huang Y, Wan C, Wu G (2020). Acute kidney injury after a stroke: a PRISMA-compliant meta-analysis. Brain Behav.

[19] Qureshi AI, Aslam H, Zafar W (2020). Acute kidney injury in acute ischemic stroke patients in clinical trials. Crit Care Med.

[20] Wang D, Guo Y, Zhang Y, Li Z, Li A, Luo Y (2018). Epidemiology of acute kidney injury in patients with stroke: a retrospective analysis from the neurology ICU. Intern Emerg Med.

